# Epidemiological profile of patients undergoing non-operative management of solid organ injury and associated factors with mortality

**DOI:** 10.1590/0100-6991e-20243734-en

**Published:** 2024-05-02

**Authors:** LUCAS MANSANO SARQUIS, IWAN AUGUSTO COLLAÇO, EDIMAR LEANDRO TODERKE, HECTOR SBARAINI FONTES, ANDRÉ THA NASSIF, ALEXANDRE COUTINHO TEIXEIRA DE FREITAS

**Affiliations:** 1 - Universidade Federal do Paraná, Clínica Cirúrgica - Curitiba - PR - Brasil; 2 - Complexo Hospitalar do Trabalhador, Cirurgia Geral e Cirurgia do Trauma - Curitiba - PR - Brasil

**Keywords:** Conservative Treatment, Abdominal Injuries, Wounds and Injuries, Trauma Centers, Tratamento Conservador, Traumatismos Abdominais, Ferimentos e Lesões, Centros de Traumatologia

## Abstract

**Introduction:**

Trauma primarily affects the economically active population, causing social and economic impact. The non-operative management of solid organ injuries aims to preserve organ function, reducing the morbidity and mortality associated with surgical interventions. The aim of study was to demonstrate the epidemiological profile of patients undergoing non-operative management in a trauma hospital and to evaluate factors associated with mortality in these patients.

**Methods:**

This is a historical cohort of patients undergoing non-operative management for solid organ injuries at a Brazilian trauma reference hospital between 2018 and 2022. Included were patients with blunt and penetrating trauma, analyzing epidemiological characteristics, blood transfusion, and association with the need for surgical intervention.

**Results:**

A total of 365 patients were included in the study. Three hundred and forty-three patients were discharged (93.97%), and the success rate of non-operative treatment was 84.6%. There was an association between mortality and the following associated injuries: hemothorax, sternal fracture, aortic dissection, and traumatic brain injury. There was an association between the need for transfusion and surgical intervention. Thirty-eight patients required some form of surgical intervention.

**Conclusion:**

The profile of patients undergoing non-operative treatment consists of young men who are victims of blunt trauma. Non-operative treatment is safe and has a high success rate.

## RESUMO

**Introdução:**

O trauma atinge principalmente a população economicamente ativa, causando impacto social e econômico. O tratamento não operatório das lesões de órgãos sólidos tem como objetivo preservar a função do órgão, diminuindo a morbimortalidade envolvida nos tratamentos cirúrgicos. O objetivo do estudo foi demonstrar o perfil epidemiológico dos pacientes submetidos ao tratamento não operatório em um hospital de trauma, bem como avaliar o fatores associados ao óbito nesses pacientes.

**Métodos:**

Trata-se de uma coorte histórica de pacientes submetidos à tratamento não operatório de lesão de órgãos sólidos, em um hospital referência de trauma brasileiro, entre 2018 e 2022. Foram incluídos pacientes vítimas de trauma contuso e penetrante, analisando as características epidemiológicas, hemotransfusão e associação ou não com necessidade de abordagem cirúrgica.

**Resultados:**

Foram incluídos 365 pacientes no estudo. Trezentos e quarenta e três pacientes receberam alta (93,97%) e a taxa de sucesso no tratamento não operatório foi de de 84,6%. Houve associação entre o desfecho óbito e as seguintes lesões associadas: hemopneumotorax, fratura de esterno, dissecção de aorta e traumatismo crânio encefálico. Houve associação entre necessidade de transfusão e abordagem cirúrgica. Trinta e oito pacientes necessitaram de alguma abordagem cirúrgica.

**Conclusão:**

O perfil dos pacientes submetidos a TNO são homens jovens, vítimas de trauma contuso. O tratamento não operatório é seguro e apresenta alta taxa de sucesso.

## INTRODUCTION

Trauma is the leading cause of death in the first four decades of life. It is responsible for high morbidity and mortality and has a greater social and economic impact than cardiovascular disease and cancer combined^1^ ^,^ ^2^.

In the context of polytrauma patients, abdominal trauma is one of the most prevalent and can cause lesions in hollow or solid viscera, such as the liver, spleen, kidney, and pancreas^3^. The nonoperative management (NOM) of abdominal solid organ lesions (SOL) in patients with hemodynamic stability has become the method of choice in the last decade, whether for blunt or penetrating trauma^2^ ^,^ ^4^. 

Trauma scores are indicators that aim to predict mortality of trauma patients. The ISS (Injury Severity Scores) evaluates the severity of the anatomical injuries, the trauma being considered if it is greater than 15^5^. The RTS (Revised Trauma Score) evaluates the physiological components related to trauma and predicts the risk of death^6^. The TRISS (Trauma and Injury Severity Scores), on its turn, determines the probability of surviving, using ISS and RTS data, as well as the type of trauma (blunt or penetrating) and the patient’s age^7^.

Currently, the indication of NOM is more related to the patient’s stability and absence of signs of peritonitis than to the American Association for the Surgery of Trauma (AAST) classification for the abdominal lesions^8^ ^-^ ^11^. Even polytrauma patients with associated injuries (extremity fractures, thoracic injuries, traumatic brain injury) may be candidates to NOM^12^ ^-^ ^14^. However, the presence of lesions in unidentified hollow viscera can lead to longer hospital stays and increased mortality^3^ ^,^ ^15^.

The objective of this study is to describe the epidemiological profile of patients undergoing NOM in a trauma hospital, as well as to evaluate the factors associated with death in these patients. 

## METHODS

This is a historical cohort, through the retrospective analysis of the electronic medical records of patients undergoing NOM of SOL at the Worker’s Hospital, a trauma referral hospital in Curitiba, State of Paraná (PR), Brazil, between 2018 and 2022. 

The study was approved by the Ethics Committee of the institution under CAAE number 24051019.1.0000.5225

We included patients with blunt or penetrating trauma and solid organ injury. Patients who underwent surgical treatment less than six hours after hospital admission were excluded.

All included patients underwent contrast-enhanced computed tomography at hospital admission for diagnosis of SOL and classification according to the AAST. The presence of lesions in the liver, spleen, right and/or left kidney, and pancreas was then assessed. We also evaluated the number of solid organs affected, as well as the presence of other associated lesions. Follow-up tests performed were not evaluated, since they were not routinely performed, being indicated by the attending team individually, according to the patient’s clinical evolution.

We analyzed sex, age, mechanism of trauma (blunt or penetrating), need or not for blood transfusion upon hospital admission, length of hospital and ICU (Intensive Care Unit) stays, need for surgical approach, and outcome (discharge or death). We also calculated the trauma indices RTS, ISS, and TRISS. 

Patients with organ lesions who remained on NOM alone were independently evaluated, as well as patients who underwent surgical treatment. After this division, the outcome (discharge or death) was assessed, with further computing of the NOM’s success rate, i.e., those patients who did not require a surgical approach and were discharged from the hospital. Based on this, we carried out a comparative analysis of these groups in relation to the variables mentioned above.

We evaluated surgeries performed on patients who failed NOM. We also analyzed whether the presence of hollow viscera lesions, the need for peritoneostomy, and ICU admission were associated with death. Moreover, we assessed the time to start chemical prophylaxis of venous thromboembolism and the relationship with the time to indicate surgical treatment.

For the statistical analysis, we initially performed a descriptive analysis of the data with estimation of the mean, median, standard deviation, and interquartile range of the quantitative variables, and simple (n) and relative (%) frequencies of the qualitative ones. We verified the association between the variables with the chi-square test. We tested the differences between groups with the Mann-Whitney and Kruskal-Wallis tests, should the variables not display a normal distribution, according to the Shapiro-Wilk test. The significance level used was 5% and all analyses were performed with the R 4.0.4 software^16^. 

## RESULTS

We included 365 patients in the study, of whom 292 (80%) had lesions in only one organ, and 73 (20%) had lesions in two or more organs. The lesions and their incidence according to the AAST classification are described in Table 1. The associated lesions identified are described in Table 2. 

**Table 1:** Frequency of lesions according to the AAST classification.

	Grade I	Grade II	Grade III	Grade IV	Grade V	Grade VI	n
Affected organs							
1	-	-	-	-	-	-	292 (80%)
2 or more	-	-	-	-	-	-	73 (20%)
Liver Injury	14 (8,6%)	52 (32,1%)	67 (41,4%)	27 (16,7%)	2 (1,2%)	0	162 (100%)
Splenic Injury	16 (10,1%)	75 (47,5%)	50 (31,7%)	17 (10,7%)	0	-	158 (100%)
Kidney Injury - Right	11 (16,2%)	33 (48,5%)	11 (16,2%)	13 (19,1%)	0	-	68 (100%)
Kidney Injury - Left	4 (8,5%)	18 (38,3%)	19 (40,4%)	6 (12,8%)	0	-	47 (100%)
Pancreatic injury	1 (11,1%)	8 (88,9%)	0	0	-	-	9 (100%)

**Table 2:** Frequency of associated injuries.

Associated injuries	n
Fracture of extremities	140 (38,3%)
Fracture of costal arches	130 (35,6%)
Traumatic brain injury	129 (35,3%)
Hemothorax and/or pneumothorax	127 (34,8%)
Pulmonary contusion	62 (17%)
Spine fracture	57 (15,6%)
Sternum fracture	8 (2,1%)
Aortic dissection	7 (1,9%)
Placental abruption	1 (0,3%)
Absence of associated lesions	86 (23,6%)

Among the included patients, 289 were male (79.2%) and 76 were female (20.8%), with a mean age of 32.4 ± 14.5 years (Table 3); 343 patients were discharged (93.9%), while 22 died (6.1%). Among the individuals who died, 15 (68.1%) did from causes unrelated to SOL, while in seven (31.9%) the cause was related to the abdominal trauma. NOM was exclusively applied to 227 patients (89.5%), while 38 (10.5%) required some type of approach. The success rate of nonoperative treatment was 85.47%. Blunt trauma was the mechanism in 345 (94.52%) patients, while 20 (5.48%) were victims of penetrating injuries. All victims of penetrating trauma were discharged, while the 22 patients who died were victims of blunt trauma. Mean RTS was 7.49, mean ISS, 16.33, and mean TRISS, 74.88 (Table 3). Patients remained hospitalized for an average of 8.4 ± 7.0 days, and 216 (59.2%) remained hospitalized only in the ward. ICU admission was required for 149 patients (40.8%), with a mean duration of 8.55 ± 9.0 days. Transfusion of blood products was necessary in 66 patients (18.1%). There was no statistical difference between the presence of lesions in two or more organs and mortality (p=0.088). However, for patients with two or more injured organs, there was a greater need of transfusion of blood products (p=0.002).

**Table 3:** Patients undergoing NOM and the outcomes discharge and death.

	General	NOM	NOM-Discharge	NOM-Death	p-value*
Sample	365	327	312 (95,4%)	15 (4,6%)	
Sex					
Male	289 (79,2%)	256 (78,3%)	245 (78,6%)	11 (73,3%)	0,63
Female	76 (20,8%)	71 (21,8%)	67 (21,4%)	4 (26,7%)	
Mean Age	32,4 ± 14,5	32,1 ± 14,3	31,6 ± 13,5	42,3 ± 23,9	0,18
Mechanism of Trauma					
Penetrating	20 (5,5%)	16 (4,9%)	16 (5,1%)	0	1,0
Blunt	345 (94,5%)	311 (95,1%)	296 (94,9%)	15 (100%)	
Trauma scores (mean)					
RTS	7,49	7,49	7,57	5,85	0,007
ISS	16,33	16,26	15,97	22,46	<0,0001
TRISS	74,88	75,12	76,20	52,76	0,03
Transfusion of blood products					
Yes	66 (18,1%)	53 (16,2)	48 (15,4%)	5 (33,3%)	0,6539
No	299 (81,9%)	274 (83,8%	264 (84,6%)	10 (66,7%)	
Need for ICU					
Yes	149 (40,8%)	123 (37,6%)	109 (34,9%)	14 (93,3%)	<0,0001
No	216 (59,2%)	204 (62,4%)	203 (65,1%)	1 (6,7%)	
Average length of hospital stay	8,4 ± 7,0	7,6 ± 5,6	7,5 ± 5,3	9,9 ± 9,5	0,3974
Average length of ICU stay	8,5 ± 9,0	8,3 ± 8,6	7,9 ± 8,1	11 ± 1,6	0,4126

Among the patients who underwent NOM only (Table 3), 312 patients (95.4%) were discharged, while 15 patients (4.6%) died. There was no statistical difference between sex and NOM patients who were discharged or died (p=0.63). The mean age among the patients who were discharged and those who died was similar (31.6 ± 13.5 years vs. 42.3 ± 23.9 years, p=0.18). Among the patients who were discharged, 296 patients (94.9%) were victims of blunt trauma and 16 (5.1%) of penetrating trauma, while among the patients who died, all were victims of blunt trauma, with no statistical significance (p=1.0). Regarding the trauma scores for patients undergoing NOM, we found a statistical significance for the three scores analyzed when comparing patients who were discharged from those who died: RTS 7.57 vs. 5.85 (p=0.007); ISS 15,97 vs. 22.46 (p<0.0001); TRISS 76.20 vs. 52.76 (p=0.03). There was no difference in blood product transfusion, which was required in 84.6% of the patients who were discharged and in 66.7% of those who died (p=0.65). On the other hand, there was an association between the need for ICU and the outcome, and for the group that was discharged, 109 patients (34.9%) were referred to the ICU and 14 (93.3%) for the patients in the subgroup who died (p<0.0001). The groups were similar for length of stay in the ward and in the ICU: 7.5 ± 5.3 vs. 9.9 ± 9.5 (p=0.3974); 7.9 ± 8.1 vs. 11 ± 1.6 (p=0.4126). There was an association between mortality and hemopneumothorax (p=0.015), sternum fracture (p<0.001), aortic dissection (p=0.015), and traumatic brain injury (TBI) (p=0.004) (Table 4).

**Table 4:** Associated lesions in patients undergoing NOM who were discharged or died.

Associated injury	NOM-Discharge	NOM-Death	p-value*
Hemothorax or pneumothorax			
No	210 (67,3%)	5 (33,4%)	0,015
Yes	102 (32,7%)	10 (66,7%)	
Fracture of costal arches			
No	201 (64,4%)	8 (53,4%)	0,549
Yes	111 (35,6%)	7 (46,6%)	
Sternum fracture			
No	307 (98,4%)	12 (80%)	<0,001
Yes	5 (1,6%)	3 (20%)	
Pulmonary contusion			
No	259 (83,1%)	11 (73,3%)	0,537
Yes	53 (16,9%)	4 (26,7%)	
Associated injury	NOM-Discharge	NOM-Death	p-value*
Limbs fracture			
No	191 (61,2%)	8 (53,4%)	0,733
Yes	121 (38,8%)	7 (46,6%)	
Spine fracture			
No	261 (83,6%)	12 (80%)	0,986
Yes	51 (16,4%)	3 (20%)	
Aortic dissection			
No	308 (98,7%)	13 (88,7%)	0,015
Yes	4 (1,3%)	2 (13,3%)	
Traumatic brain injury			
No	206 (66,1%)	4 (26,7%)	0,004
Yes	106 (33,9%)	11 (73,3%)	

There were 38 interventions after NOM failure (Table 5), four (10.5%) endovascular embolizations and 34 (89.5%) surgeries. Among the 34 patients who underwent surgery, six had a hollow viscus lesion, this being associated with death (p=0.012). There was also an association between death and the need for peritoneostomy (p<0.001) and ICU admission (p<0.001). The mean time for surgical approach was 4.9 ± 4.53 days for patients who were discharged and 1.86 ± 0.69 days for patients who died (p=0.1235).

**Table 5:** Patients undergoing surgical treatment and outcome.

	Discharge	Death	p-value*
Mean age (Standard deviation)	31,6 ± 13,5	44,5 ± 22,5	0,008
Approach			
Surgical	27	7	0,001
Embolization	4	0	
Hollow viscera lesion			
No	25	3	0,012
Yes	2	4	
Need for peritoneostomy after surgical approach			
No	24	1	<0,001
Yes	3	6	
Mean time to surgical approach (SD)	4,9 ± 4,53	1,86 ± 0,69	0,61

## DISCUSSION

Nonoperative management is currently the option of choice for solid organ injuries, whether blunt or penetrating, with a success rate between 78% and 98%^17^. A systematic review showed that hospitals with a higher volume of nonoperative treatment are considered an independent factor for greater NOM success (OR=2.15) and shorter hospital stay. The indication of NOM is directly linked to the patient’s hemodynamic stability and the availability of a trauma surgeon with experience in NOM than to the classification of the organ lesion according to the AAST^18^.

Computed tomography is essential for NOM planning and appropriate indication, with good sensitivity even for penetrating lesions on the dorsum or in the thoraco-abdominal transition and may also make use of rectal and/or oral contrast in selected cases^19^ ^,^ ^20^. Although the recent implementations of hybrid rooms have shown an improvement in the care of polytrauma patients^21^, this is not the reality of most trauma services in Brazil. We believe that it is possible to perform NOM in almost all trauma centers in the country, due to the evolution of CT scanners and the ability of trauma surgeons to interpret exams, even for patients with TBI or other associated injuries^22^.

Patients undergoing NOM in the present study had a mean ISS of 16.26, showing that even in severe cases with high-grade lesions, treatment may be indicated, although these are associated with greater NOM failure. Most patients were admitted with RTS greater than 7, suggesting a high probability survival, between 98.8% and 96.9%^6^. Considering that in most abdominal injuries there is no change in the level of consciousness at hospital admission, the RTS value of this group of patients may be overestimated. When we analyzed the patients who underwent NOM and died, we found the lowest mean RTS (mean of 5.85), which was shown to be a predictor of NOM failure and death. The mean TRISS of the sample was 75.12, but when we evaluated only the patients who died, it was 52.76, reinforcing the good structure of care for trauma patients^24^. Trauma scores have limitations, but the identification of the largest number of factors that can lead to NOM failure is essential to improve its outcomes. 

In the present study, there was no significant difference in relation to age and patients who were discharged or died. Despite the lower incidence of NOM in the elderly, with the highest life expectancy in the population, trauma in the elderly has become increasingly frequent and challenging. The presence of comorbidities or the use of medications may be related to higher in-hospital mortality after trauma^25^ ^,^ ^26^.

Associated injuries can lead to NOM failure, as well as increase in the length of hospital stay and the need for ICU^12^. In the present series, extremity fractures were the most prevalent associated injuries (38.3%). There was an association between the outcomes and hemothorax and/or pneumothorax, sternum fracture, aortic dissection, and TBI. These lesions identify high-energy traumas, which can be a hampering factor for the good evolution of NOM. One of the fears of applying NOM is the suspicion of hollow viscera lesions, because the late diagnosis of such injuries can cause diffuse peritonitis, sepsis, and organ dysfunctions^15^ ^,^ ^27^. This fact was demonstrated in this study through the association between the presence of hollow viscera lesions and death. Despite the risk of associated lesions causing NOM failure, there was no statistical significance for the outcome when comparing the presence of injury in only one organ or in two or more organs in NOM patients. However, the presence of two or more affected organs showed a greater need for blood transfusion. There was an association between the need for blood transfusion and the surgical approach. Several studies indicate that the need for blood products increases the risk of NOM failure^4^ ^,^ ^20^ ^,^ ^28^, but there was no such association in the present study.

With the passage of time and the experience of surgeons, NOM has shown positive results when well indicated for patients who are victims of penetrating trauma^20^. A retrospective study with 501 patients evaluated the cost of treating isolated abdominal penetrating lesions, showing a mean cost of £410 (pounds sterling) for NOM, £780 for non-therapeutic exploratory laparotomy, and £870 for diagnostic laparoscopy^29^. In the present study, all patients with penetrating lesions were discharged, confirming good results when well indicated, in addition to bringing lower cost to the service when compared to non-therapeutic surgeries.

The spleen is the main organ injured in blunt trauma^22^. However, in the present study, it had the second highest incidence. NOM was initiated with the intention of avoiding splenectomy in children and preserving the organ’s immune function^30^. Over time, this approach was extended to all ages. Isolated lesions of the spleen have a good response to NOM, with up to 96.8% success rate^27^ ^,^ ^29^. However, the higher the injury’s degree, the greater the risk of failure, which can reach up to 75% for grade 5 lesions^13^ ^,^ ^23^. Larger splenic lesions (grade III or IV) may be conducted by NOM, but attention should be paid to the risk of late bleeding, as well as the need for endovascular embolization^32^. 

The main factors related to NOM failure in splenic lesions are ISS greater than 15, age greater than 55 years, associated liver injury, contrast extravasation in the arterial phase, and need for four or more packed red blood^13^ ^,^ ^22^. In the present study, splenectomy was necessary in 12 cases, but all of them were discharged, suggesting that the early identification of NOM failure had no impact on outcome.

Another concern related to NOM in patients with larger splenic lesions is the risk of delayed bleeding. Late rupture occurs on average 48 to 72 hours after trauma, especially in the presence of pseudoaneurysms or subcapsular hematoma^33^. A retrospective analysis of 6,857 patients identified late rupture in 32 (0.4%) who had normal CT scans on admission. However, after specialists reviewed the images, 71% of these CT scans were considered of poor quality, preventing the correct diagnosis at admission^34^. Fractures of the lower costal arches and injury to other solid organs were present in 40% of the patients with late rupture^35^.

With the advancement of endovascular techniques, embolization has become an alternative to control bleeding. However, a retrospective study with 37,000 splenic embolizations showed a higher incidence of infection after one year in patients undergoing embolization when compared with patients undergoing NOM or even splenectomy^36^. While the Western Trauma Association suggests embolization in the presence of tomographic blush^37^, the Eastern Association for the Surgery of Trauma (EAST) advises that the patient’s clinical condition should be evaluated, suggesting that control tests be performed within 72 hours to assess the real need for embolization^38^.

The success rate of NOM for liver injuries ranges from 74% to 94%, and the risk of failure increases with higher ISS, greater degree of injury, or lower RTS^1^. There is a higher mortality rate in patients undergoing surgical treatment compared to NOM for severe hepatic trauma^2^. In a retrospective Brazilian study, the failure rate for hepatic NOM was 11.36%, associated with patients with high ISS or multiple blunt traumas. In the same study, the main cause of death was related to TBI and not to complications of hepatic trauma^15^.

Hepatic complications of NOM, such as biliary fistulas or perihepatic collections, can be treated by interventional radiology or laparoscopy^1^, the most common one being perihepatic collection (3.1%), followed by biliary fistula (1.5%)^39^. In the present study, laparoscopy for drainage of biliary collection was performed in five patients, with therapeutic success after their hospital discharge. Due to the severity of the liver injury, five cases required hepatic packing and peritoneostomy, and of these, three died, reinforcing the literature that shows the morbidity and mortality involved in patients treated surgically in severe liver trauma^2^. Complications such as pseudoaneurysm, arteriovenous fistula, and abdominal compartment syndrome have an incidence of less than 1%^39^, though the only hepatic embolization performed in the present study was in a patient with penetrating hepatic trauma with pseudoaneurysm of a branch of the left hepatic artery. 

Pancreatic trauma has a high mortality rate, but with a relatively low incidence (0.4%-2%) and an associated duodenal injury should be suspected due to anatomical proximity^40^ ^,^ ^41^. When pancreatic duct lesions are suspected, magnetic resonance imaging can confirm the diagnosis, and is essential for the correct indication of a surgical approach, evaluating the topography of the lesion (head or body and tail)^41^. In our series, none of the patients had duct or hollow viscera lesions associated with pancreatic trauma, but two individuals required peri-pancreatic drainage due to suspicion of infected collection.

Renal injury may be present in 1% to 5% of traumas, and NOM is the treatment of choice to preserve kidney function and reduce the morbidity involved in nephrectomy, especially in the long term^42^. NOM is safe even for penetrating kidney injuries. Only two patients with penetrating kidney injury required selective embolization of the renal artery, one performed on the third and the other on the tenth day of hospitalization, both due to pseudoaneurysm. If the patient undergoes exploratory laparotomy for other reasons, it is still possible to perform renal NOM without exploration of the retroperitoneum, because the opening of the Gerota’s fascia can uncover the hematoma, leading to a higher risk of nephrectomy^43^.

Some questions are often raised among surgeons during the management of NOM. Is there a need for repeated imaging? When is the ideal time to start chemical prophylaxis for venous thromboembolism? How long is a hospital stay required for safe hospital discharge?

The need for follow-up imaging tests can be done routinely or directed according to the patient’s clinical evolution. In this study, we did not analyze the control tests performed, but the routine of the service is that only patients with clinical worsening or persistent drop in hemoglobin levels should be submitted to control tests. A retrospective study with 365 patients with liver injury found that 59% of patients underwent control tomography only in the presence of clinical alterations and identified no statistical difference for late complications or the need for intervention^44^. However, some studies suggest its need after 72 hours for cases of grade IV or V liver lesions^45^. For renal lesions, imaging is indicated between 48 and 72 hours for patients with alterations in the clinical picture or for those with lesions of the collecting system on admission tomography^42^. On the other hand, for splenic lesions, some authors suggest routinely performing follow-up examinations, abdominal ultrasound, or contrast-enhanced tomography for high-grade lesions (grades III or IV)^45^ ^,^ ^46^, given the higher risk of late bleeding, especially due to underdiagnosed subcapsular hematoma^33^. 

Teichman et al.^47^ compared absolute rest and early ambulation to hematimetric stability in patients with splenic or hepatic trauma. There was a decrease in the length of hospital stay in patients with early ambulation, with no increase in the rate of NOM failure. This is also the recommendation of the Consensus of the World Society of Emergency Surgery, where patients with minor lesions are encouraged to ambulate early from admission, while individuals with larger lesions are released for ambulation according to the absence of a drop greater than 10% in hemoglobin on the first day^30^.

Early ambulation also decreases the risk of venous thromboembolism (VTE), which can be present in up to 4.5% of patients undergoing NOM^48^. A systematic review of 4,642 patients observed a higher risk of NOM failure with the early introduction of chemical prophylaxis, but without increasing the need for blood transfusion^49^. However, another study, with 36,000 patients, evaluated the introduction of chemical prophylaxis for VTE within 48 hours of trauma and observed a lower rate of VTE, with no difference in the need for blood transfusion, incidence of NOM failure, or mortality^50^. Complications such as deep vein thrombosis (DVT) are lower in groups with early introduction of chemical prophylaxis for VTE (up to 48 hours after trauma), but with no repercussion on mortality^49^. In the present study, only one patient with NOM failure had chemical prophylaxis started before the surgical approach. However, in this case NOM failure was related to a hollow viscus injury and not to bleeding.

A randomized study^28^ evaluated the ideal time for hospital discharge in patients undergoing NOM, comparing discharge on the third or fifth days of hospitalization. These patients were victims of hepatic or splenic injury, with a mean ISS of 16, and were followed up for 30 days after discharge. It observed that the period of greatest risk for failure was in the first 72 hours after trauma, and discharge on the third day was safe. Other authors suggest that the five-day period would be ideal for managing splenic lesions due to the risk of late rupture^31^. In our study, the mean length of hospital stay was 8.3 ± 8.6 days and was mainly related to the presence of associated lesions and not to the management of the SOL itself. 

## CONCLUSION

The profile of patients undergoing NOM are young men victims of blunt force trauma. The factors associated with death in patients undergoing NOM were hemopneumothorax, sternum fracture, traumatic brain injury, aortic dissection, severe trauma according to the RTS, ISS, and TRISS scores, and the need for ICU admission.

## References

[1] Brooks A, Reilly J, Hope C (2020). Evolution of non-operative management of liver trauma. Trauma Surg Acute Care Open.

[2] Park KB, You DD, Hong TH, Heo JM, Won YS (2015). Comparison between operative versus non-operative management of traumatic liver injury. Korean J Hepatobiliary Pancreat Surg.

[3] Chu M, How N, Laviolette A, Bilic M, Tang J, Khalid M (2022). Delayed laparoscopic peritoneal washout in non-operative management of blunt abdominal trauma a scoping review. World J Emerg Surg.

[4] Cretcher M, Panick CEP, Boscanin A, Farsad K (2021). Splenic trauma endovascular treatment approach. Ann Transl Med.

[5] Baker SP, O'Neill B (1974). The injury severity score a method for describing patients with multiple injuries and evaluating emergency care. J Trauma.

[6] Champion HR (1989). A Revision of the Trauma Score. J Trauma.

[7] Boyd CR, Tolson MA (1987). Evaluating trauma care the TRISS method. Trauma Score and the Injury Severity Score. J Trauma.

[8] Coccolini F, Montori G, Catena F, Kluger Y, Biffl W, Moore EE (2017). Splenic trauma WSES classification and guidelines for adult and pediatric patients. World J Emerg Surg.

[9] Coccolini F, Coimbra R, Ordonez C (2020). Liver trauma WSES 2020 guidelines. World J Emerg Surg.

[10] Coccolini F, Kobayashi L, Kluger Y (2019). Duodeno-pancreatic and extrahepatic biliary tree trauma WSES-AAST guidelines. World J Emerg Surg.

[11] Coccolini F, Moore EE, Kluger Y (2019). Kidney and uro-trauma WSES-AAST guidelines. World J Emerg Surg.

[12] Teuben MPJ, Spijkerman R, Blokhuis TJ, Pfeifer R, Teuber H, Pape HC (2018). Safety of selective nonoperative management for blunt splenic trauma the impact of concomitant injuries. Patient Saf Surg.

[13] Meira JD, Menegozoo CAM, Rocha MC, Utiyama EM (2021). Non-operative management of blunt splenic trauma evolution, results and controversies. Rev Col Bras Cir.

[14] McIntyre LK, Schiff M, Jurkovich G (2005). Failure of Nonoperative Management of Splenic Injuries Causes and Consequences. Arch Surg.

[15] Fernandes GS, Martins MC, Gomes HL (2023). Experience of non-operative management of blunt liver trauma at Hospital das Clínicas de Uberlândia 114 cases. Rev Col Bras Cir.

[16] R Core Team (2021). R: A language and environment for statistical computing.

[17] Zenaidi H, Ismail IB, Rebii S, Zoghlami A (2020). Predictors of Failure of Nonoperative Management in Spleen Trauma. J Emerg Trauma Shock.

[18] Lavanchy JL, Delafontaine L, Haltmeier T, Bednarski P, Schnüriger B (2022). Increased hospital treatment volume of splenic injury predicts higher rates of successful non-operative management and reduces hospital length of stay a Swiss Trauma Registry analysis. Eur J Trauma Emerg Surg.

[19] Martins EL, Mazepa MM, Guetter CR, Pimentel SK (2018). The role of computerized tomography in penetrating abdominal trauma. Rev Col Bras Cir.

[20] Habashi R, Coates A, Engels PT (2019). Selective nonoperative management of penetrating abdominal trauma at a level 1 Canadian trauma centre a quest for perfection. Can J Surg.

[21] Loftus TJ, Croft CA, Rosenthal MD, Mohr AM, Efron PA, Moore FA (2021). Clinical Impact of a Dedicated Trauma Hybrid Operating Room. J Am Coll Surg.

[22] Yildiz A, Özpek A, Topçu A, Yücel M, Ezberci F (2022). Blunt splenic trauma Analysis of predictors and risk factors affecting the non-operative management failure rate. Ulus Travma Acil Cerrahi Derg.

[23] Carvalho FH, Romeiro PC, Collaço IA, Baretta GA, Freitas AC, Matias JE (2009). Prognostic factors related to non surgical treatment failure of splenic injuries in the abdominal blunt trauma. Rev Col Bras Cir.

[24] Costa CD da S, Scarpelini S (2012). Avaliação da qualidade do atendimento ao traumatizado através do estudo das mortes em um hospital terciário. Rev Col Bras Cir.

[25] Gioffrè-Florio M, Murabito LM, Visalli C, Pergolizzi FP, Famà F (2018). Trauma in elderly patients a study of prevalence, comorbidities and gender differences. G Chir.

[26] Eryurt SC, Sahin T, Oral S (2023). Evaluation of factors affecting prognosis and mortality in geriatric patients presented to the emergency service with head trauma. Aging Med (Milton).

[27] Cunha SC, DE-Oliveira AG, Miranda ML (2023). Analysis of the efficacy and safety of conservative treatment of blunt abdominal trauma in children retrospective study. Conservative treatment of blunt abdominal trauma in children. Rev Col Bras Cir.

[28] Kumar V, Mishra B, Joshi MK, Purushothaman V, Agarwal H, Anwer M (2021). Early hospital discharge following non-operative management of blunt liver and splenic trauma A pilot randomized controlled trial. Injury.

[29] Dayananda K, Kong VY, Bruce JL, Oosthuizen GV, Laing GL, Clarke DL (2017). Selective non-operative management of abdominal stab wounds is a safe and cost effective strategy A South African experience. Ann R Coll Surg Engl.

[30] Djordjevic I, Zivanovic D, Budic I, Kostic A, Djeric D (2021). Importance of a Follow-Up Ultrasound Protocol in Monitoring Posttraumatic Spleen Complications in Children Treated with a Non-Operative Management. Medicina (Kaunas).

[31] Bagaria D, Kumar A, Ratan A, Gupta A, Kumar A, Kumar S (2019). Changing Aspects in the Management of Splenic Injury Patients Experience of 129 Isolated Splenic Injury Patients at Level 1 Trauma Center from India. J Emerg Trauma Shock.

[32] Podda M, De Simone B, Ceresoli M, Virdis F, Favi F, Wiik Larsen J (2022). Follow-up strategies for patients with splenic trauma managed non-operatively the 2022 World Society of Emergency Surgery consensus document. World J Emerg Surg.

[33] Romeo L, Andreotti D, Lacavalla D, Ferro S, Tondo M, Salviato E (2020). Delayed Rupture of a Normal Appearing Spleen After Trauma Is Our Knowledge Enough? Two Case Reports. Am J Case Rep.

[34] Harmon L, Bilow R, Shanmuganathan K (2019). Delayed splenic hemorrhage Myth or mystery? A Western Trauma Association multicenter study. Am J Surg.

[35] Fugazzola P, Morganti L, Coccolini F. (2020). The need for red blood cell trans- fusions in the Emergency Department as a risk factor for failure of non-op- erative management of splenic trauma A multicenter prospective study. Eur J Trauma Emerg Surg.

[36] Cioci AC, Parreco JP, Lindenmaier LB, Olufajo OA, Namias N, Askari R (2019). Readmission for infection after blunt splenic injury A national comparison of management techniques. J Trauma Acute Care Surg.

[37] Bhullar IS, Frykberg ER, Tepas JJ, Siragusa D, Loper T, Kerwin AJ (2013). At first blush absence of computed tomography contrast extravasation in grade IV or V adult blunt splenic trauma should not preclude angioembolization. J Trauma Acute Care Surg.

[38] Skattum J, Naess PA, Eken T, Gaarder C (2013). Refining the role of splenic angiographic embolization in high-grade splenic injuries. J Trauma Acute Care Surg.

[39] Virdis F, Podda M, Di Saverio S, Kumar J, Bini R, Pilasi C (2022). Clinical outcomes of non-operative management and clinical observation in non-angioembolised hepatic trauma A systematic review of the literature. Chin J Traumatol.

[40] Matias N, Jegatheeswaran S, Nadarajah V, Sheen AJ, Jamdar S, Siriwardena AK (2020). Non-operative management of pancreatic trauma in adults. Hepatobiliary Pancreat Dis Int.

[41] Girard E, Abba J, Cristiano N, Siebert M, Barbois S, Létoublon C (2016). Management of splenic and pancreatic trauma. J Visc Surg.

[42] Kelly CE, Bowers KE, Holton AE, Van Embden D (2022). Non-operatively managed blunt and penetrating renal trauma Does early follow up CT scan change management? A systematic review. Injury.

[43] Clements TW, Ball CG, Nicol AJ, Edu S, Kirkpatrick AW, Navsaria P (2022). Penetrating renal injuries an observational study of non-operative management and the impact of opening Gerota's fascia. World J Emerg Surg.

[44] Fletcher KL, Perea LL, Morgan ME, Otaibi BW, Hazelton JP (2021). Repeat Imaging in Blunt Hepatic Injuries Can Wait for Clinical Change. J Surg Res.

[45] Kanlerd A, Auksornchart K, Boonyasatid P (2022). Non-operative management for abdominal solidorgan injuries A literature review. Chin J Traumatol.

[46] Cartu D, Margaritescu D, Sandulescu S, Bratiloveanu T, Ramboiu S, Bica M (2021). Nonoperative Treatment of Abdominal Trauma Involving Liver and Spleen. Chirurgia (Bucur).

[47] Teichman A, Scantling D, McCracken B, Eakins J (2018). Early mobilization of patients with non-operative liver and spleen injuries is safe and cost effective. Eur J Trauma Emerg Surg.

[48] Joseph B, Pandit V, Harrison C, Lubin D, Kulvatunyou N, Zangbar B (2015). Early thromboembolic prophylaxis in patients with blunt solid abdominal organ injuries undergoing nonoperative management is it safe?. Am J Surg.

[49] Lamb T, Lenet T, Zahrai A, Shaw JR, McLarty R, Shorr R (2022). Timing of pharmacologic venous thromboembolism prophylaxis initiation for trauma patients with nonoperatively managed blunt abdominal solid organ injury a systematic review and meta-analysis. World J Emerg Surg.

[50] Alejandro KV, Acosta JA, Rodríguez PA (2003). Bleeding manifestations after early use of low-molecular- weight heparins in blunt splenic injuries. Am Surg.

